# Baltimore College of Dental Surgery
*This notice was overlooked at its proper place.


**Published:** 1860-04

**Authors:** 


*Baltimore College of Dental Surgery.**—The Twentieth Annual Commencement of this Institution was held on the evening of February 28th. On that occasion the following gentlemen received the degree of Doctor of Dental Surgery, with the usual ceremonies :

| | | Subjects of Theses. |
|---|---|---|
| Amzi W. Alexander, | N. C. | Extracting Teeth. |
| James B. Bean, . | Fla. | Pivot Teeth. |
| Thomas E. Bessellieu, . | S. C. | Dental Etiquette. |
| Charles Billingslea, . | Md. | Effects of Diseased Teeth. |
| John Bland, . . . | Ark. | Inflammation of the Mouth. |
| Hugh P. Bone, . . | Ala. | Inflammation. |
| Memory Bonner, M. D., | S. C. | The Saliva. |
| Abiel Bowen, . . | N. Y. | Preparation for Artificial Dentures. |
| Conrad S. Boyd, . . | N. C. | Teeth and their Diseases. |
| William N. Cunningham, | La. | Irregularity of Teeth. |
| Wm. B. Dennis, . . | R. I. | Primary Dentition. |
| John H. Dickson, M. D., | S. C. | The Natural Teeth. |
| John W. Doniphan, M. D., | S. C. | Fractures. |
| Charles E. Dunn, . | Ky. | Mechanical Dentistry. |
| Wm. C. Dunnavant, . | Va. | Dental Education. |
| William H. Gates, . | Ga. | Filling Teeth. |
| Charles O. Hall, . . | Conn. | Vulcanite Basis. |
| Thaddeus Haynie, . | Va. | Filling Teeth. |
| Thomas M. Henly, . . | Va. | Caries and its Treatment. |
| J. Carroll House, . | N. Y. | Impressions and Metallic Dies. |
| P. Fernando Hyatt, . | Penn. | Odontalgia. |
| Theodore A. Lafar, . | S. C. | Ætiology of Odontalgia. |
| John M. Lazier, . . | Va. | Salivary Calculus. |
| George C. De Marini, | R. I. | Salivary Glands. |
| David R. McCallum, . | S. C. | Dental Education. |
| Adam H. May, . . | Penn. | Organs of Mastication. |
| John S. Moore, . . | N. C. | Extraction of Teeth. |
| Henry H. Nelles, . . | Canada. | Odontography. |
| Perry Ould, . . | Md. | Mechanical Dentistry. |
| George Paterson, . . | Ga. | Ætiology of Odontalgia. |
| Daniel E. Provost, . | N. Y. | Odontology. |
| C. Dighton Seward, . | Texas. | Teeth and their Diseases. |
| J. Vernon Simmons, . | Md. | Tic Douloureux. |
| Archibald Small, . . | N. C. | Dental Mechanism. |
| Benjamin Smith, . | Md. | Dental Caries. |
| Samuel Stoddard, . . | Va. | The Animal Kingdom. |
| B. S. Traywick, . | N. C. | Inserting Pivot Teeth. |
| William H. Waters, . | Vt. | Neurology. |
| C. A. Woodward, . | R. I. | Development of the Teeth. |

* This notice was overlooked at its proper place.

After the conferring the degrees, Professor Bond delivered the valedictory.

It may be gratifying to the friends of the College to state that the last class—numbering seventy students—was the largest ever in attendance at any course of dental instruction.

The prize offered by Dr. Mallet, of Connecticut, was awarded to Geo. C. De Marini, for his thesis on "The Salivary Glands." It will be found in another part of the present number of the Journal, illustrated by Dr. De Marini from dissections made by him at the College during the past winter.

Ten other theses, of those presented in competition for the prize, were mentioned by the Committee of Award as highly creditable.

